# Epigenetic Modification of Death Receptor Genes for TRAIL and TRAIL Resistance in Childhood B-Cell Precursor Acute Lymphoblastic Leukemia

**DOI:** 10.3390/genes12060864

**Published:** 2021-06-05

**Authors:** Atsushi Watanabe, Kunio Miyake, Koshi Akahane, Kumiko Goi, Keiko Kagami, Hideo Yagita, Takeshi Inukai

**Affiliations:** 1 Department of Pediatrics, Faculty of Medicine, University of Yamanashi, Yamanashi 409-3898, Japan; awatanabe@yamanashi.ac.jp (A.W.); akoushi@yamanashi.ac.jp (K.A.); kgoi@yamanashi.ac.jp (K.G.); kkagami@yamanashi.ac.jp (K.K.); 2 Department of Health Sciences, University of Yamanashi, Yamanashi 409-3898, Japan; kmiyake@yamanashi.ac.jp; 3 Department of Immunology, School of Medicine, Juntendo University, Tokyo 113-8421, Japan; hyagita@juntendo.ac.jp

**Keywords:** TNF-related apoptosis-inducing ligand, death receptors, epigenetics, B-cell precursor acute lymphoblastic leukemia

## Abstract

Immunotherapies specific for B-cell precursor acute lymphoblastic leukemia (BCP-ALL), such as anti-CD19 chimeric antigen receptor (CAR) T-cells and blinatumomab, have dramatically improved the therapeutic outcome in refractory cases. In the anti-leukemic activity of those immunotherapies, TNF-related apoptosis-inducing ligand (TRAIL) on cytotoxic T-cells plays an essential role by inducing apoptosis of the target leukemia cells through its death receptors (DR4 and DR5). Since there are CpG islands in the promoter regions, hypermethylation of the *DR4* and *DR5* genes may be involved in resistance of leukemia cells to immunotherapies due to TRAIL-resistance. We analyzed the *DR4* and *DR5* methylation status in 32 BCP-ALL cell lines by sequencing their bisulfite PCR products with a next-generation sequencer. The *DR4* and *DR5* methylation status was significantly associated with the gene and cell-surface expression levels and the TRAIL-sensitivities. In the clinical samples at diagnosis (459 cases in the NOPHO study), both *DR4* and *DR5* genes were unmethylated in the majority of cases, whereas methylated in several cases with dic(9;20), *MLL*-rearrangement, and hypodiploidy, suggesting that evaluation of methylation status of the *DR4* and *DR5* genes might be clinically informative to predict efficacy of immunotherapy in certain cases with such unfavorable karyotypes. These observations provide an epigenetic rational for clinical efficacy of immunotherapy in the vast majority of BCP-ALL cases.

## 1. Introduction

**Figure 1:**
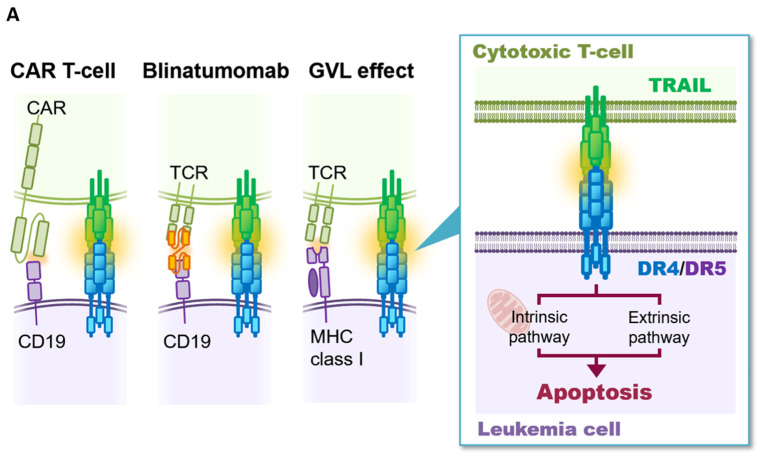

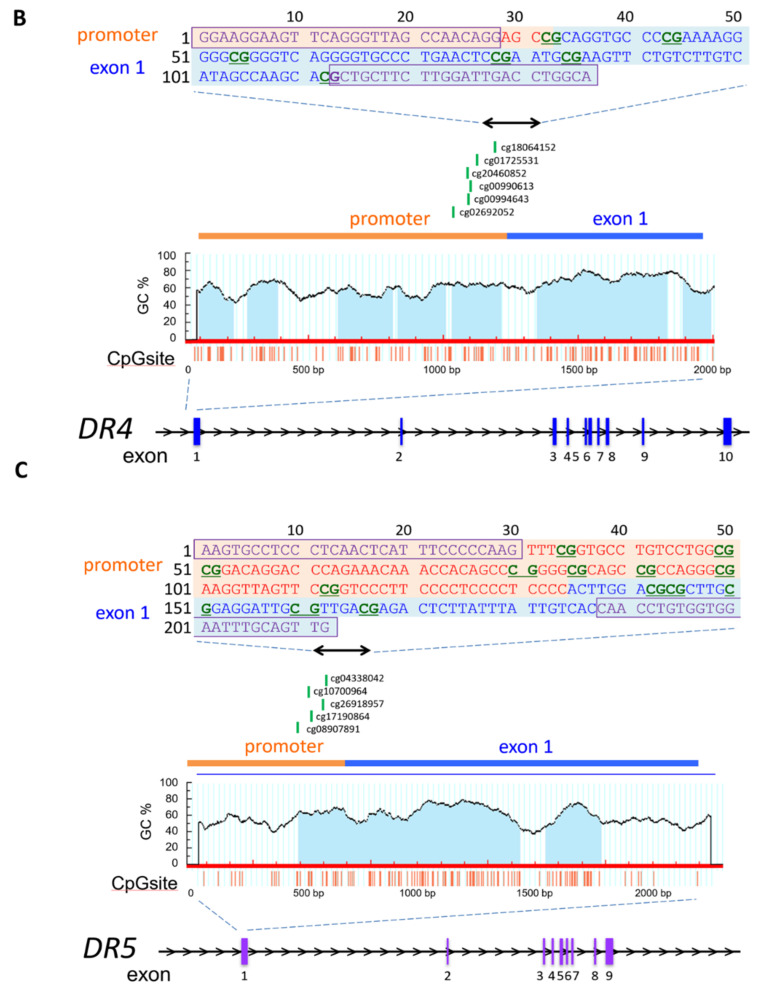

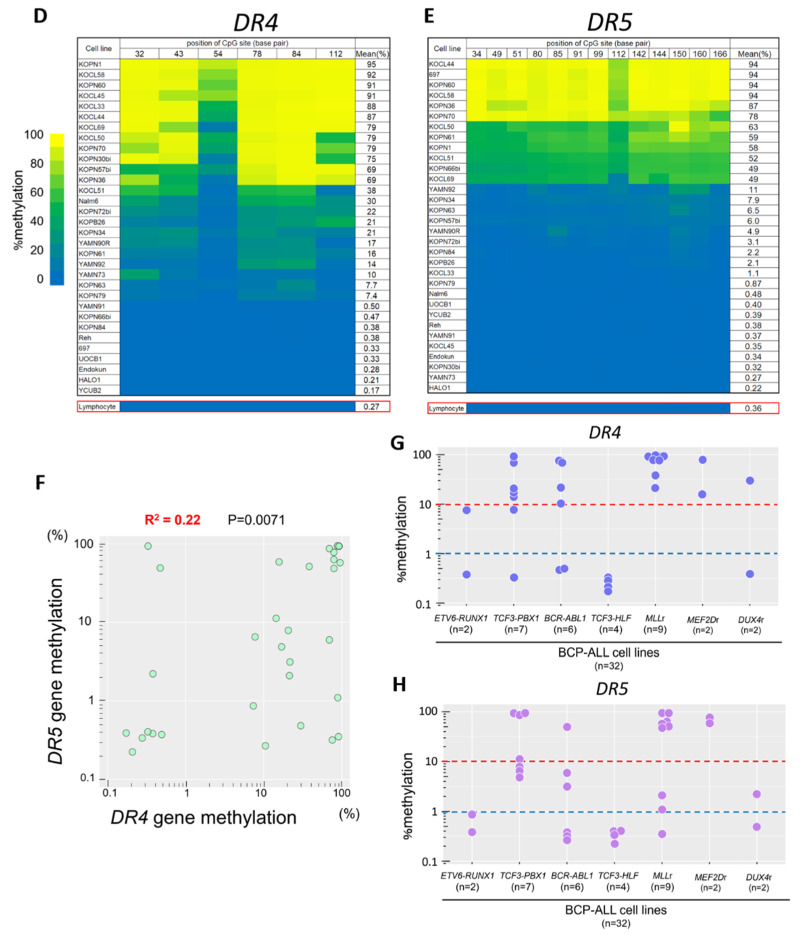
Methylation status of the *DR4* and *DR5* genes in BCP-ALL cell lines. (**A**) Schematic representation of TRAIL/death receptor (DR4 and DR5) system in anti-leukemic activity (left panel) induced by CAR T-cell (left), Blinatumomab (middle), and GVL effect (right) after allogeneic stem cell transplantation and two independent apoptotic pathways (right panel) induced by TRAIL/death receptor system. Abbreviations; CAR, chimeric antigen receptor; GVL, graft versus leukemia; TCR, T-cell receptor; MHC, major histocompatibility complex. (**B**,**C**) Schematic representation of the CpG islands in human *DR4* (**B**) and *DR5* (**C**) genes. Bisulfite PCR of the 136-bp region (containing 6 CG dinucleotides) of the *DR4* gene (**B**) and 212-bp region (containing 13 CG dinucleotides) of the *DR5* gene (**C**) was performed, and the methylation status of each CG dinucleotide was evaluated. In the top panels, sequences analyzed by bisulfite PCR are indicated. Boxes indicate primers for PCR. In the middle panels, location of each CG dinucleotide in the methylation database analyzed by the NOPHO study is indicated. (**D**,**E**) Heat map of the methylation status in each CG dinucleotide of bisulfite PCR products of the *DR4* (**D**) and *DR5* (**E**) genes in representative BCP-ALL cell lines. In the bottom, the methylation status in the peripheral lymphocytes from a healthy volunteer is indicated. (**F**), Correlation between the methylation status of the *DR4* and that of the *DR5* in 32 BCP-ALL cell lines. Horizontal and vertical axes indicate a log_10_ percent methylation of the *DR4* and that of the *DR5*, respectively. *R*^2^ and *p*-value in Spearman’s rank correlation coefficient are indicated at the top of the panel. (**G**,**H**) Association of the methylation status of the *DR4* (**G**) and *DR5* (**H**) genes with representative karyotypes in 32 BCP-ALL cell lines.

Two recently established immunotherapies specific for B-cell precursor acute lymphoblastic leukemia (BCP-ALL) have dramatically improved the therapeutic outcome in poor prognostic BCP-ALL patients. One is chimeric antigen receptor (CAR) T-cell therapy targeting at CD19 [1], and the other is a CD3/CD19-engaging antibody, blinatumomab [2]. Both therapies are mediated by anti-leukemic activity of activated T-cells targeting at CD19 molecules expressed on BCP-ALL cells. In addition to these immunotherapies, cytotoxic T-cells (CTLs), which are critically involved in graft-versus leukemia (GVL), are affected after allogeneic stem cell transplantation (allo-SCT) for the patients with poor prognostic leukemia [3,4,5]. CTLs induce apoptotic cell death into residual leukemia cells through cytotoxic factors. Among cytotoxic factors, TNF-related apoptosis-inducing ligand (TRAIL) plays a role in the GVL effect [6,7]. TRAIL is expressed on the surface of natural killer cells and CTLs and binds to its death receptors expressing on the surface of leukemia cells [8,9]. There are two types of death receptors for TRAIL: DR4 (TNFRSF10A, TRAIL-R1, CD261) [10] and DR5 (TNFRSF10B, TRAIL-R2, CD262) [11]. In bone marrow transplantation models using TRAIL-deficient mice, TRAIL is essential for optimal graft-versus-tumor activity by donor T cells, while it plays little or no role in the development of graft-versus-host disease [6,7]. We previously demonstrated that *BCR-ABL1*-positive leukemia cells, which are clinically sensitive to the GVL effect [12] frequently express DR4 and/or DR5, and, subsequently, are sensitive to anti-leukemic activity of recombinant human soluble TRAIL (rhsTRAIL) [13]. In contrast, *MLL* (*KMT2A*)-rearranged (*MLL*r) acute lymphoblastic leukemia (ALL) cells, which are clinically resistant to the GVL effect [14], generally express very low or undetectable levels of DR4 and DR5, and, subsequently, are resistant to rhsTRAIL [15]. We also confirmed that cell-surface expression of TRAIL on CAR T-cells is upregulated in the co-culture with targeted BCP-ALL cells [16]. Moreover, recent CRISPR screens identified TRAIL receptor as a key mediator of anti-CD19 CAR T-cell cytotoxicity against BCP-ALL [17]. Accordingly, expression of DR4 and/or DR5 is a critical factor in the susceptibility of leukemia cells to the anti-leukemic activity of TRAIL and consequently to immunotherapies using the CAR T-cells and blinatumomab as well as the GVL effect after allo-SCT (Figure 1A).

Hypermethylation of the CpG island in the gene promoter is an epigenetic modification of gene expression. We previously reported that cell lines and patients’ samples of T-cell ALL (T-ALL) showed a TRAIL resistance in association with low cell-surface expression levels of DR4 and DR5 [18]. Semi-quantitative analysis using methylation-specific PCR revealed that the methylation status of the gene promoter in T-ALL cell lines was associated with the gene expression level, at least for *DR4* [18]. These observations suggest that the methylation status of the *DR4* and/or *DR5* genes may be associated with the DR4 and DR5 expression and consequently with the TRAIL sensitivity in BCP-ALL cells. However, to date, little is known about the relevance of the epigenetic modification of the *DR4* and *DR5* genes in BCP-ALL.

In the present study, we quantified the methylation status of the CpG islands of the *DR4* and *DR5* gene promoter in BCP-ALL cell lines by sequencing their bisulfite PCR products with a next-generation sequencer (NGS). We found that hypermethylation of the *DR4* and *DR5* CpG islands is highly associated with a lack of cell-surface expression of DR4 and DR5, and TRAIL resistance in BCP-ALL cell lines. We further investigated the significance of the methylation status of the *DR4* and *DR5* genes in clinical samples of BCP-ALL patients.

## 2. Materials and Methods

### 2.1. Leukemia Cell Lines and Patients’ Samples

Nine *MLL*r-ALL cell lines (KOPN1, KOPB26, KOCL33, KOCL44, KOCL45, KOCL50, KOCL51, KOCL58, and KOCL69) [15]; six *BCR-ABL1*-positive ALL cell lines (KOPN30bi, KOPN57bi, KOPN 66bi, KOPN 72bi, YAMN73, and YAMN91) [13]; seven *TCF3-PBX1*-positive ALL cell lines (697, KOPN34, KOPN36, KOPN60, KOPN63, YAMN90, and YAMN92); four *TCF3-HLF*-positive ALL cell lines (YCUB2, Endo-kun, UOC-B1, and HAL-O1) [18]; and two *ETV6-RUNX1*-positive ALL cell lines (KOPN79 and Reh); two *MEF2D*r-ALL cell lines (KOPN61 and KOPN70); and two *DUX4*r-ALL cell lines (KOPN84 and Nalm6) were used in this study (Supplementary Table S1). All cell lines were maintained in RPMI1640 medium supplemented with 10% fetal calf serum (FCS) in a humidified atmosphere of 5% CO_2_ at 37 °C. Forty-nine cryopreserved samples of childhood BCP-ALL (35 samples at diagnosis and 14 samples at relapse after chemotherapy) (Supplementary Table S2) were analyzed after approval by the ethics committee at the University of Yamanashi.

### 2.2. ^3^H-Thymidine Uptake Assay

Sensitivities to TRAIL were determined as previously reported [13,15,18,19] using rhsTRAIL (Killer TRAIL, San Diego, CA, USA). In brief, cells (5 × 10^4^ cells/well) were cultured in the absence or presence of 100 ng/mL of rhsTRAIL in triplicate in 200 μL of RPMI1640 medium supplemented with 10% FCS in a flat-bottomed 96-well plate. The plates were incubated for 42 h, pulsed for the last 6 hours of the incubation with ^3^H-thymidine (1 μCi/well) and harvested onto glass-fiber filters. The level of radioactivity incorporated into DNA was measured by liquid scintillation counting. The percent inhibition by rhsTRAIL was calculated as the ratio of the radioactivity to that in the absence of rhsTRAIL.

### 2.3. Cell-Surface Expression of DR4 and DR5

Cell-surface expression of DR4 and DR5 was determined as previously reported using the monoclonal antibodies (mAbs) specific to DR4 and DR5 [13,15,18,19]. Leukemia cell lines were incubated with 1 μg of biotinylated control mouse IgG_1_ or mAb on ice for 30 min. After washing, the cells were incubated with phycoerythrin-conjugated streptavidin (Biomeda, Foster City, CA, USA) on ice for 30 min, and then analyzed by flow cytometry. The relative florescence intensity (RFI) was calculated as the ratio of the mean fluorescence intensity of specific staining to that of control staining.

### 2.4. Real-Time Polymerase Chain Reaction Analysis

Gene expression levels of *DR4* and *DR5* were quantified as previously reported by real-time reverse transcription polymerase chain reaction (RT-PCR) analysis [18,19]. Total RNA was extracted using the Trizol reagent (Invitrogen, Carlsbad, CA, USA) following the manufacturer’s directions. Reverse transcription was performed with 3 μg of total RNA, random hexamer (Amersham Bioscience, Buckinghamshire, UK) and Superscript II reverse transcriptase (Invitrogen) at conditions recommended by the manufacturer, and then incubated with 1 μL of RNase (Invitrogen) at 37 °C for 20 min. For quantitative real-time RT-PCR of *DR4* and *DR5* transcripts, triplicated samples containing 9 μL of cDNA with 10 μL of Taqman Universal PCR Master Mix (Applied Biosystems, Foster City, CA, USA) and 1 μL of 20 × Assays-on-demand Gene Expression Product (*DR4*; Hs 00269492_m1, *DR5*; Hs 00366272_m1, Applied Biosystems) were pre-incubated at 50 °C for 2 min and subsequently at 95 °C for 10 min. Amplification was obtained by 40 cycles of reaction at 95 °C for 15 sec and 60 °C for 1 min. Fluorescence data were quantitatively analyzed on ABI Prism 7500 Sequence Detection System (Applied Biosystems). Nalm1, a TRAIL sensitive chronic myelogenous leukemia blast crisis -derived cell line that expresses DR4 and DR5 [13], was used for control. As internal control, quantitative real-time RT-PCR for glyceraldehyde-3-phosphate dehydrogenase (*GAPDH*; Hs 99999905_m1, Applied Biosystems) was performed.

### 2.5. Bisulfite Sequencing

Bisulfite PCR was performed as previously reported [20]. Genomic DNA was subjected to sodium bisulfite modification with an EZ DNA Methylation-Lightning kit (Zymo Research, Irvine, CA, USA). Bisulfite modified DNA was amplified by PCR for *DR4* gene with forward primer (5′-GGAAGGAAGTTTAGGGTTAGTTAATAG-3′) and reverse primer (5′-TACCAAATCAATCCAAAAAACAAC-3′) and *DR5* gene with forward primer (5′-AAGTGTTTTTTTTAATTTATTTTTTTTAAG-3′) and reverse primer (5′-CAACTACAAATTCCACCACAAATTA-3′) using one cycle of 95 °C for 4 min, 40 cycles of 95 °C for 30 sec, 55 °C for 30 sec, 72 °C for 1 min, with a final cycle of 72 °C for 7 min. For conventional Sanger sequencing, PCR products were cloned into a pTAC-2 vector using a TA PCR Cloning Kit (BioDynamics, Tokyo, Japan) and sequenced. For next-generation sequencing, amplicon libraries were generated by Ion Plus Fragment Library Kit (MAN0006846, Thermo Fisher Scientific, Waltham, MA, USA) and Ion Xpress Barcode Adaptors Kit (Thermo Fisher Scientific). Briefly, 50 ng amplicon was end-repaired, nick-repaired, and Ion Torrent adapters P1 and Barcode were ligated with DNA ligase. Following Agencourt AMPure XP purification (Beckman Coulter, Brea, CA, USA), the individual libraries were quantified by quantitative real-time PCR then diluted, and finally pooled in equimolar ratios. The libraries were processed with the Ion OneTouch™ 2 System using Ion PGM™ Template OT2 400 Kit (Thermo Fisher Scientific) to produce 400 base-read libraries. Sequencing was performed by an Ion PGM™ Hi-Q Sequencing Kit (Thermo Fisher Scientific) using 850 flows on the Ion 318 Chip Kit v2 (Thermo Fisher Scientific) according to the manufacturer’s protocol. After sequencing, single processing and base-calling were performed using Torrent Suite 5.0.2 (Thermo Fisher Scientific). Methylation analysis was performed using MethylationAnalysis_Amplicon plug-in v1.3 (Thermo Fisher Scientific).

### 2.6. Gene Methylation and Gene Expression Analyses in Childhood BCP-ALL Cohort

Gene methylation of childhood BCP-ALL clinical samples were investigated using 450 k DNA methylation array database of Nordic Pediatric Hematology and Oncology (NOPHO) (Gene Expression Omnibus with accession number; GSE49031) [21]. Among 764 ALL samples at diagnosis, the data of 459 BCP-ALL cases with following representative chromosomal aberrations were analyzed; high-hyperdiploid (51–67 chromosomes [22]), *ETV6-RUNX1*, *TCF3-PBX1*, iAMP21, dic(9;20), *BCR-ABL1*, *MLL*r, hypodiploid (<45 chromosomes), and polyploid (>67 chromosomes). The data of the paired samples at diagnosis and at relapse were also analyzed in 24 relapsed BCP-ALL cases. Percent methylation of *DR4* and *DR5* was evaluated by mean methylation level of CG dinucleotides that were annotated as both 0–200 bases upstream of the transcriptional start site (TSS200) and CpG island using Illumina HumanMethylation450 BeadChip (GPL13534; Illumina Inc., San Diego, CA, USA) [21].

### 2.7. Statistics

Mann–Whitney U test, Fisher’s exact test, Chi-square test, and Spearman’s correlation analysis were performed using R software version 3.5.2 (R Core Team 2018).

## 3. Results

### 3.1. Methylation Status of CpG Islands in the DR4 and DR5 Genes in BCP-ALL Cell Lines

There are typical CpG islands in boundary regions between the promoter and exon 1 of the *DR4* (Figure 1B) and *DR5* (Figure 1C) genes. To investigate their methylation status, we performed bisulfite PCR of a 136-bp region (containing 6 CG dinucleotides) of the *DR4* gene (Figure 1B) and a 212-bp region (containing 13 CG dinucleotides) of the *DR5* gene (Figure 1C) using specific primers that contain no CG dinucleotide. We quantified the methylation level of each CG dinucleotide by sequencing bisulfite PCR products of 32 BCP-ALL cell lines using NGS. In the CpG island of the *DR4* (Figure 1D) and *DR5* (Figure 1E) genes, the methylation level of each of the 6 CG dinucleotides and the 13 CG dinucleotides, respectively, was almost similar in each cell line. Mean percent methylation of 6 CG dinucleotides of the *DR4* gene and 13 CG dinucleotides of the *DR5* gene varied from 0% to 94% among 32 cell lines. As a whole, a weak but a significant positive correlation (*R*^2^ = 0.22, *p* = 0.0071) was observed between the mean percent methylation of the *DR4* gene and that of the *DR5* gene in 32 cell lines (Figure 1F). In normal lymphocytes from a healthy volunteer, both the *DR4* and *DR5* genes were unmethylated analyzed using bisulfite NGS method revealed 0.27% and 0.36%, respectively (Figure 1D,E).

We next analyzed an association of the methylation status of the *DR4* and *DR5* genes with karyotypes in BCP-ALL cell lines. The *DR4* gene was methylated (>10%) in all of nine *MLL*r-ALL and two *MEF2D*r-ALL cell lines, while it was unmethylated (<1%) in all of four *TCF3-HLF*-positive ALL cell lines (Figure 1G). A similar trend was observed in the methylation status of the *DR5* gene (Figure 1H). The *DR5* gene was methylated (>10%) in six of nine *MLL*r-ALL cell lines and all of two *MEF2D*r-ALL cell lines, while it was unmethylated in all of four *TCF3-HLF*-positive ALL and two *ETV6-RUNX1*-positive ALL cell lines. Despite being limited to a small number of cell lines, these observations suggest that methylation status of the *DR4* and *DR5* genes may be associated with karyotypes of BCP-ALL cell lines.

### 3.2. Association of the Methylation Status of the DR4 and the DR5 with Their Gene and Cell-Surface Expressions and rhsTRAIL Sensitivity in BCP-ALL Cell Lines

**Figure 2:**
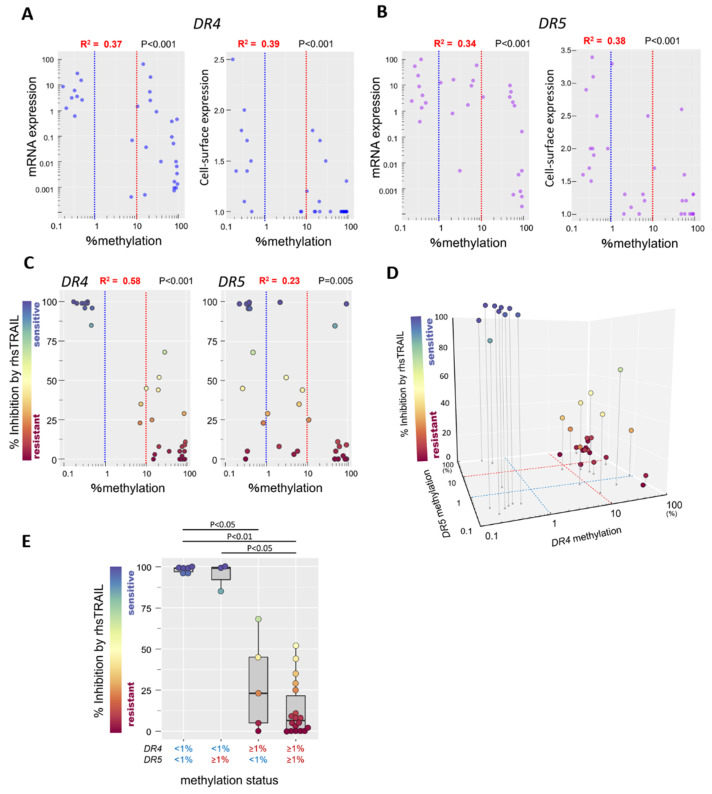
Association of the methylation status with gene and cell-surface expression of the *DR4* and *DR5* and TRAIL sensitivity in BCP-ALL cell lines. (**A**,**B**) Correlation of methylation status with gene and cell-surface expression of the *DR4* (**A**) and *DR5* (**B**) in 32 BCP-ALL cell lines. Horizontal axes indicate a log_10_ percent methylation of the *DR4* and *DR5* genes. Vertical axes indicate gene (left panels) and cell-surface (right panels) expression levels of the *DR4* (**A**) and *DR5* (**B**). *R*^2^ and *p*-value in Spearman’s rank correlation coefficient are indicated at the top of the panel. (**C**) Correlation of the methylation status of the *DR4* and *DR5* genes with rhsTRAIL sensitivity in 32 BCP-ALL cell lines. Horizontal axes indicate a log_10_ percent methylation of the *DR4* (left panel) and the *DR5* (right panel). Vertical axes indicate a percent inhibition by rhsTRAIL (100 ng/mL). *R*^2^ and *p*-value in Spearman’s rank correlation coefficient are indicated at the top of the panel. (**D**) Three-dimensional representation of rhsTRAIL inhibition and the methylation status of *DR4*/*DR5* genes. Horizontal axis and vertical axis indicate log_10_ percent methylation of the *DR4* and *DR5* genes, respectively. (**E**) TRAIL sensitivity of BCP-ALL cell lines with different methylation status of the *DR4* and *DR5* genes. Vertical axis represents a percent inhibition by rhsTRAIL (100 ng/mL). *p*-values in Mann–Whitney U test are indicated at the top of the panel. In (**C**–**E**) TRAIL-sensitivity in each cell line is also indicated with color-scale.

We previously reported that cell-surface expression levels of DR4 and DR5 are significantly correlated with their gene expression levels in BCP-ALL cell lines [13,15,19]. Thus, we investigated an association between the methylation status of the *DR4* and *DR5* genes and the gene and cell-surface expression levels of DR4 and DR5 in 32 BCP-ALL cell lines. Mean percent methylation of *DR4* gene showed a significant negative correlation, both with the gene expression level of *DR4* (*R*^2^ = 0.37) and with the cell-surface expression level of DR4 (*R*^2^ = 0.39) (Figure 2A). Similarly, mean percent methylation of the *DR5* gene showed a significant negative correlation, both with the gene expression level of the *DR5* gene (*R*^2^ = 0.34) and the cell-surface expression level of DR5 (*R*^2^ = 0.38) (Figure 2B).

Next, we examined an association of the methylation status of the *DR4* and *DR5* genes with the sensitivity to anti-leukemic activity of rhsTRAIL [13,15,19]. Mean percent methylation of the *DR4* gene showed a significant negative correlation with the percent inhibition by rhsTRAIL (*R*^2^ = 0.58) (Figure 2C). To compare with the *DR4* gene, the association of the *DR5* gene methylation status with the rhsTRAIL sensitivity was less significant; correlation coefficient (*R*^2^) between mean percent methylation of the *DR5* gene and percent inhibition by rhsTRAIL was 0.23 (Figure 2C). Since both DR4 and DR5 have a death domain, either DR4 or DR5 cell-surface expression may be sufficient for TRAIL sensitivity. Thus, we performed a three-dimensional analysis of the *DR4* and *DR5* methylation status and the rhsTRAIL sensitivity (Figure 2D). Of note, three *DR4*-preferentially unmethylated cell lines (mean percent methylation; *DR4* <1%, *DR5* ≥1%) (697, KOPN84, and KOPN66bi) as well as six unmethylated cell lines (*DR4* and *DR5* <1%) (Reh, YAMN91, Endo-kun, HAL-O1, UOC-B1, and YCUB2) were highly sensitive to rhsTRAIL (percent inhibition ≥80%). In contrast, three of five *DR5*-preferentially unmethylated cell lines (*DR4* ≥1%, *DR5* <1%) as well as 13 of 18 methylated cell lines (*DR4* and *DR5* ≥1%) were resistant to rhsTRAIL (percent inhibition <25%). Among them, all of the 11 highly methylated cell lines (*DR4* and *DR5* ≥10%) were resistant to rhsTRAIL (percent inhibition <25%). These observations indicate that the methylation status of *DR4* and *DR5* (particularly *DR4*) is tightly associated with rhsTRAIL sensitivity in BCP-ALL cell lines (Figure 2E).

### 3.3. Low Methylation Status of CpG Islands in the DR4 and DR5 Genes in ALL Samples

**Figure 3:**
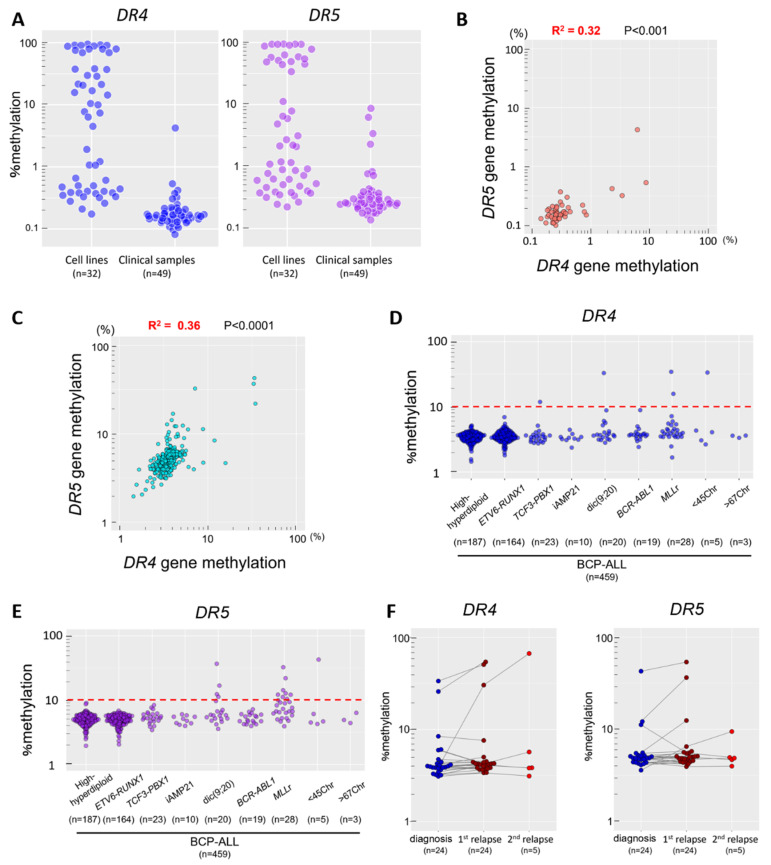
Methylation status of the *DR4* and *DR5* genes in clinical samples of childhood BCP-ALL. (**A**) Comparison of the methylation status of the *DR4* and *DR5* genes between the cell lines and the clinical samples. Vertical axes indicate a log_10_ percent methylation of the *DR4* (left panel) and the *DR5* (right panel) genes determined by sequencing of bisulfite PCR products in 32 cell lines and 49 clinical samples from BCP-ALL. (**B**,**C**) Correlation between the methylation status of the *DR4* and that of the *DR5* in the 49 clinical samples (**B**) and in 459 clinical samples at diagnosis from childhood BCP-ALL with representative karyotypes of in the NOPHO cohort (**C**). Horizontal and vertical axes indicate a log_10_ percent methylation of the *DR4* and that of the *DR5*, respectively. *R*^2^ and *p*-value in Spearman’s rank correlation coefficient are indicated at the top of the panel. (**D**,**E**) Association of the *DR4* (**D**) and *DR5* (**E**) methylation status with karyotypes in the 459 BCP-ALL samples at diagnosis in the NOPHO cohort study. Vertical axes represent a log_10_ percent methylation of the *DR4* and *DR5* genes, respectively. (**F**) Changes in the methylation levels of the *DR4* and *DR5* genes in 24 paired samples at diagnosis and at relapse in the NOPHO cohort study. Vertical axes indicate a log_10_ percent methylation of the *DR4* (left panel) and that of the *DR5* (right panel).

We next examined the methylation status of the *DR4* and *DR5* genes in 49 childhood BCP-ALL samples by sequencing bisulfite PCR-product using the NGS. In contrast to cell lines, mean percent methylation levels of the *DR4* and *DR5* genes were less than 1% in the majority of the samples (Figure 3A). Only one (2%) and four (8%) samples were weakly methylated (1–10%) in the *DR4* and *DR5* genes, respectively. Although the relevance of correlation is limited due to largely unmethylated status, a weak positive correlation (*R*^2^ = 0.32, *p* < 0.001) was observed between the mean percent methylation of the *DR4* gene and that of the *DR5* gene in 49 samples (Figure 3B). In the 49 clinical samples, 35 samples were obtained at diagnosis whereas 14 samples were at relapse after chemotherapy (Supplementary Table S2). Among 35 samples at diagnosis, 24 and 11 samples were classified into standard and high-risk groups of the National Cancer Institute (NCI) criteria, respectively. The percent methylation of the *DR4* and *DR5* genes was not upregulated in the samples in high-risk group at diagnosis (Supplementary Figure S1A) and the samples at relapse (Supplementary Figure S1B). Next, we extensively evaluated mean percent methylation of six and five CG dinucleotides annotated to the promoter of the *DR4* and *DR5* genes and located in the TSS200 region, respectively, using the genome wide DNA methylation data in a large childhood BCP-ALL cohort study of NOPHO (GSE49031) [21]. A significant positive correlation (*R*^2^ = 0.35, *p* < 0.001) was observed between the percent methylation of the *DR4* gene and that of the *DR5* gene in 459 BCP-ALL samples (Figure 3C). In comparison with the above results in the NGS analysis, baseline levels of methylation were relatively higher in methylation array database of the NOPHO study. In the majority of the samples, mean percent methylation of both the *DR4* and the *DR5* genes was less than 10%. Only five (1.1%; 1 of 23 *TCF3-PBX1*-positive; 1 of 20 dic(9;20)-positive; 2 of 28 *MLL*r-positive; 1 of 5 hypodiploid) ALL samples (Figure 3D) and 16 (3.5%; 5 of 20 dic(9;20)-positive; 10 of 28 *MLL*r-positive; and 1 of 5 hypodiploid) ALL samples (Figure 3E) showed a relatively higher methylation level (≥10%) in the *DR4* and the *DR5* genes, respectively. Both the *DR4* and *DR5* genes were unmethylated (<10%) in all of 187 high hyperdiploid ALL, 164 *ETV6-RUNX1*-positive ALL, 10 iAMP21-positive ALL, and 19 *BCR-ABL1*-positive ALL samples. In the bone marrow samples in complete remission and normal T-cells and B-cells, % methylation of the *DR4* and *DR5* genes were approximately 2–3%. In the majority of samples from patient at relapse, the percent methylation of *DR4* and *DR5* genes was almost unchanged compared to that of diagnosis. However, in the paired samples at diagnosis and at relapse, methylation levels of *DR4* and *DR5* genes were upregulated in several cases at relapse (Figure 3F). These observations suggest that the *DR4* and *DR5* genes are largely unmethylated in BCP-ALL clinical samples, particularly in the samples with the favorable karyotypes such as hyperdiploidy and *ETV6-RUNX1* fusion and those with certain high risk/poor prognostic karyotypes such as *BCR-ABL1* and iAMP21.

## 4. Discussion

In the present study, we quantitatively evaluated the methylation status of the CpG islands in the *DR4* and *DR5* genes by using a BCP-ALL cell line as a model system. In BCP-ALL cell lines, the methylation status of the *DR4* and *DR5* genes was associated with their mRNA and cell-surface expression levels and their rhsTRAIL sensitivities, indicating that epigenetic modification of the *DR4* and *DR5* genes due to hypermethylation is a mechanism for TRAIL resistance in BCP-ALL. However, we observed discrepancies in the methylation status of the *DR4* and *DR5* genes between the cell lines and the clinical samples. The *DR4* and the *DR5* genes were not methylated in the majority of clinical samples. This difference in methylation status may be partly attributed to the different distribution of karyotypes between the cell lines and clinical samples. In BCP-ALL cell lines, the *DR4* and *DR5* genes were unmethylated in *ETV6-RUNX1*-positive ALL cell lines, while being frequently methylated in *MLL*r-ALL cell lines. Similarly, in the genome-wide DNA methylation data of BCP-ALL samples in the NOPHO cohort, the *DR4* and *DR5* genes were exclusively unmethylated in the samples with *ETV6-RUNX1* but relatively frequently methylated in the samples with *MLL*r. In our series of BCP-ALL cell lines, only 6% (2/32) of cell lines had *ETV6-RUNX1*, while 31% (10/32) of cell lines showed the *MLL*r karyotype. In contrast, in the NOPHO cohort, over one-third (164/459) of the samples had *ETV6-RUNX1*, while only 6% (28/459) of the samples showed the *MLL*r karyotype. We also observed upregulation of the *DR4*/*DR5* methylation status in several relapsed cases (Figure 3F). In this context, it should be noted that 20 out of 32 cell lines (Supplementary Table S1) were established from the samples at relapse. Thus, higher methylation levels of the *DR4* and *DR5* genes in cell lines may be partly attributed to the fact that two thirds of our cell lines were established from the samples at relapse. In case of patient samples, the samples may contain certain numbers of normal hematopoietic cells. In this context, we confirmed that the *DR4* and *DR5* genes were generally unmethylated in bone marrow samples in complete remission and in normal peripheral lymphocytes (Supplementary Figure S2). Thus, the methylation level in the clinical samples may be underestimated due to a contamination of unmethylated normal cells. Finally, it has been previously reported that, although cancer cell lines retained methylation status of their tumor of origin, CpG island hypermethylation was more prominent in cell lines than in original cancer tissues [23]. Our observations seem to be consistent with this previous finding.

As a new therapeutic modality, immunotherapy using anti-CD19 CAR T-cells and blinatumomab is promising for refractory BCP-ALL cases. In a recent genome-wide CRISPR-Cas9 screen of Nalm6, a BCP-ALL cell line, *DR5*, was identified as one of key mediators of anti-CD19 CAR T-cell cytotoxicity [17]. In our analysis, Nalm6 is one of *DR5*-preferentially unmethylated (mean percent methylation; *DR4* 30%, *DR5* 0.5%) cell lines and is moderately sensitive to rhsTRAIL (percent inhibition; 68%), suggesting that the *DR5* gene, but not the *DR4* gene, plays an essential role in CAR T-cell cytotoxicity against Nalm6 due to unmethylated status of the *DR5* gene. Meanwhile, we previously reported that cell-surface expression of TRAIL on anti-CD19 CAR T-cells is upregulated by the co-culture with targeted BCP-ALL cells [16]. These observations indicated that the TRAIL/death receptor system mediates anti-leukemic activity of anti-CD19 CAR T-cells against BCP-ALL.

Blinatumomab is a bispecific T-cell engager antibody simultaneously binding CTLs and CD19-positive BCP-ALL cells [24], suggesting that TRAIL/death receptor system may also be involved in anti-leukemic activities of blinatumomab. In the present study, it was clearly demonstrated that gene silencing due to hypermethylation of the *DR4* and *DR5* genes is associated with rhsTRAIL resistance of BCP-ALL cell lines. However, gene silencing of the *DR4* and *DR5* genes due to hypermethylation was uncommon in the majority of BCP-ALL cases at diagnosis. Accordingly, resistance to immunotherapy due to TRAIL resistance as a result of hypermethylation of the *DR4* and *DR5* genes is unlikely in the majority of BCP-ALL cases, at least at disease onset. In several relapsed cases of the NOPHO cohort study, upregulation was observed in the methylation status of the *DR4* and *DR5* genes. Although the precise treatment in these cases was unknown, this observation suggests the possibility that acquired hypermethylation of the *DR4* and/or *DR5* genes may be one of mechanisms for relapse particularly after immunotherapy.

Of importance, in the analysis of clinical samples, hypermethylation of the *DR4* and/or *DR5* genes was observed in certain karyotypes such as dic(9;20), *MLL*r, and hypodiploidy. Thus, when the BCP-ALL patients with these karyotypes are treated by immunotherapy, methylation status and/or gene/cell-surface expression levels of the *DR4* and *DR5* genes might be useful biomarkers to predict therapeutic responses. In this context, we previously reported that *TCF3-HLF*-positive ALL cells are highly sensitive to TRAIL, since *TCF3-HLF* fusion transcription factor effectively transactivates the *DR4* and *DR5* gene expression [19]. Indeed, in the present study, the *DR4* and *DR5* genes are unmethylated in all of four *TCF3-HLF*-positive ALL cell lines. *TCF3-HLF*-positive ALL is the most unfavorable type of childhood BCP-ALL due to resistance to conventional chemotherapy [25,26]. Of note, recent combination therapy of blinatumomab with allo-SCT successfully induces a durable remission in the majority of *TCF3-HLF*-positive ALL patients resistant to a conventional chemotherapy [27]. Our findings provide an additional epigenetic rationale for blinatumomab in *TCF3-HLF*-positive ALL patients.

## 5. Conclusions

The present study revealed that TRAIL-resistance due to hypermethylation of the *DR4* and *DR5* genes is unlikely in the majority of BCP-ALL cases, particularly in the cases with favorable karyotypes such as hyperdiploidy and *ETV6-RUNX1*. Since TRAIL/death receptor system plays an essential role in the anti-leukemic activities of immunotherapy using anti-CD19 CAR T-cells, our findings provide an epigenetic rationale for clinical efficacy of immunotherapy in BCP-ALL patients. Moreover, in certain BCP-ALL cases with unfavorable karyotypes such as dic (9;20), MLLr, and hypodiploidy, evaluation of methylation status of the *DR4* and *DR5* genes might be clinically informative to predict the efficacy of immunotherapy.

## Data Availability

The data presented in this study are available on request from the corresponding author. The open dataset used in our manuscript are available in the Gene Expression Omnibus database (https://www.ncbi.nlm.nih.gov/geo/) (accessed on 5 June 2021).

## Supplementary Materials

The following are available online at https://www.mdpi.com/article/10.3390/genes12060864/s1. Figure S1: (A), Comparison of the methylation status of the *DR4* and *DR5* genes between the BCP-ALL samples at diagnosis in NCI standard risk group (*n* = 24) and those in high risk group (*n* = 11). Vertical axes indicate a log_10_ percent methylation of the *DR4* (left panel) and the *DR5* (right panel) genes. P-values in Mann-Whitney’s U test are indicated at the top of the panel. (B), Comparison of the methylation status of the *DR4* and *DR5* genes between the BCP-ALL samples at diagnosis (*n* = 35) and those at relapse (*n* = 11). Vertical axes indicate a log_10_ percent methylation of the *DR4* (left panel) and the *DR5* (right panel) genes. Lines indicate the changes in the four paired samples. P-values in Mann-Whitney’s U test are indicated at the top of the panel. Figure S2: The *DR4* and *DR5* methylation status in BCP-ALL samples at diagnosis (*n* = 459), bone marrow (BM) samples in complete remission (CR) (*n* = 86), and normal peripheral T-cells (*n* = 25) and B-cells (*n* = 25) in the NOPHO database. Vertical axes indicate a log_10_ percent methylation of the *DR4* (left panel) and that of the *DR5* (right panel). Table S1: Characteristics of BCP-ALL cell lines; Table S2: Characteristics of BCP-ALL clinical samples.

## References

[1] Maude S.L., Laetsch T.W., Buechner J., Rives S., Boyer M., Bittencourt H., Bader P., Verneris M.R., Stefanski H.E., Myers G.D. (2018). Tisagenlecleucel in Children and Young Adults with B-Cell Lymphoblastic Leukemia. N. Engl. J. Med..

[2] Kantarjian H., Stein A., Gökbuget N., Fielding A.K., Schuh A.C., Ribera J.M., Wei A., Dombret H., Foà R., Bassan R. (2017). Blinatumomab versus Chemotherapy for Advanced Acute Lymphoblastic Leukemia. N. Engl. J. Med..

[3] Kolb H.J., Schmid C., Barrett A.J., Schendel D.J. (2004). Graft-versus-leukemia reactions in allogeneic chimeras. Blood.

[4] Singh A.K., McGuirk J.P. (2016). Allogeneic Stem Cell Transplantation: A Historical and Scientific Overview. Cancer Res..

[5] Dickinson A.M., Norden J., Li S., Hromadnikova I., Schmid C., Schmetzer H., Jochem-Kolb H. (2017). Graft-versus-Leukemia Effect Following Hematopoietic Stem Cell Transplantation for Leukemia. Front. Immunol..

[6] Takeda K., Hayakawa Y., Smyth M.J., Kayagaki N., Yamaguchi N., Kakuta S., Iwakura Y., Yagita H., Okumura K. (2001). Involvement of tumor necrosis factor-related apoptosis-inducing ligand in surveillance of tumor metastasis by liver natural killer cells. Nat. Med..

[7] Smyth M.J., Cretney E., Takeda K., Wiltrout R.H., Sedger L.M., Kayagaki N., Yagita H., Okumura K. (2001). Tumor necrosis factor-related apoptosis-inducing ligand (TRAIL) contributes to interferon γ-dependent natural killer cell protection from tumor metastasis. J. Exp. Med..

[8] Schmaltz C., Alpdogan O., Kappel B.J., Muriglan S.J., Rotolo J.A., Ongchin J., Willis L.M., Greenberg A.S., Eng J.M., Crawford J.M. (2002). T cells require TRAIL for optimal graft-versus-tumor activity. Nat. Med..

[9] Lelaidier M., Dìaz-Rodriguez Y., Cordeau M., Cordeiro P., Haddad E., Herblot S., Duval M. (2015). TRAIL-mediated killing of acute lymphoblastic leukemia by plasmacytoid dendritic cell-activated natural killer cells. Oncotarget.

[10] Pan G., O’Rourke K., Chinnaiyan A.M., Gentz R., Ebner R., Ni J., Dixit V.M. (1997). The receptor for the cytotoxic ligand TRAIL. Science.

[11] Pan G., Ni J., Wei Y.F., Yu G., Gentz R., Dixit V.M. (1997). An antagonist decoy receptor and death domain-containing receptor for TRAIL. Science.

[12] Aricò M., Valsecchi M.G., Camitta B., Schrappe M., Chessells J., Baruchel A., Gaynon P., Silverman L., Janka-Schaub G., Kamps W. (2000). Outcome of treatment in children with Philadelphia chromosome-positive acute lymphoblastic leukemia. N. Engl. J. Med..

[13] Uno K., Inukai T., Kayagaki N., Goi K., Sato H., Nemoto A., Takahashi K., Kagami K., Yamaguchi N., Yagita H. (2003). TNF-related apoptosis-inducing ligand (TRAIL) frequently induces apoptosis in Philadelphia chromosome-positive leukemia cells. Blood.

[14] Pui C.H., Gaynon P.S., Boyett J.M., Chessells J.M., Baruchel A., Kamps W., Silverman L.B., Biondi A., Harms D.O., Vilmer E. (2002). Outcome of treatment in childhood acute lymphoblastic leukaemia with rearrangements of the 11q23 chromosomal region. Lancet.

[15] Inukai T., Zhang X., Goto M., Hirose K., Uno K., Akahane K., Nemoto A., Goi K., Sato H., Takahashi K. (2006). Resistance of infant leukemia with MLL rearrangement to tumor necrosis factor-related apoptosis-inducing ligand: A possible mechanism for poor sensitivity to antitumor immunity. Leukemia.

[16] Saito S., Nakazawa Y., Sueki A., Matsuda K., Tanaka M., Yanagisawa R., Maeda Y., Sato Y., Okabe S., Inukai T. (2014). Anti-leukemic potency of piggyBac-mediated CD19-specific T cells against refractory Philadelphia chromosome-positive acute lymphoblastic leukemia. Cytotherapy.

[17] Dufva O., Koski J., Maliniemi P., Ianevski A., Klievink J., Leitner J., Pölönen P., Hohtari H., Saeed K., Hannunen T. (2020). Integrated drug profiling and CRISPR screening identify essential pathways for CAR T-cell cytotoxicity. Blood.

[18] Akahane K., Inukai T., Zhang X., Hirose K., Kuroda I., Goi K., Honna H., Kagami K., Nakazawa S., Endo K. (2010). Resistance of T-cell acute lymphoblastic leukemia to tumor necrosis factor-related apoptosis-inducing ligand-mediated apoptosis. Exp. Hematol..

[19] Zhang X., Inukai T., Hirose K., Akahane K., Kuroda I., Honna-Oshiro H., Kagami K., Goi K., Nakamura K., Kobayashi M. (2012). Oncogenic fusion E2A-HLF sensitizes t(17;19)-positive acute lymphoblastic leukemia to TRAIL-mediated apoptosis by upregulating the expression of death receptors. Leukemia.

[20] Huang M., Miyake K., Kagami K., Abe M., Shinohara T., Watanabe A., Somazu S., Oshiro H., Goi K., Goto H. (2017). Lack of association between deletion polymorphism of BIM gene and in vitro drug sensitivity in B-cell precursor acute lymphoblastic leukemia. Leuk. Res..

[21] Nordlund J., Bäcklin C.L., Wahlberg P., Busche S., Berglund E.C., Eloranta M.L., Flaegstad T., Forestier E., Frost B.M., Harila-Saari A. (2013). Genome-wide signatures of differential DNA methylation in pediatric acute lymphoblastic leukemia. Genome Biol..

[22] Paulsson K., Johansson B. (2009). High hyperdiploid childhood acute lymphoblastic leukemia. Genes Chromosomes Cancer.

[23] Smiraglia D.J., Rush L.J., Frühwald M.C., Dai Z., Held W.A., Costello J.F., Lang J.C., Eng C., Li B., Wright F.A. (2001). Excessive CpG island hypermethylation in cancer cell lines versus primary human malignancies. Hum. Mol. Genet..

[24] Topp M.S., Kufer P., Gökbuget N., Goebeler M., Klinger M., Neumann S., Horst H.A., Raff T., Viardot A., Schmid M. (2011). Targeted therapy with the T-cell-engaging antibody blinatumomab of chemotherapy-refractory minimal residual disease in B-lineage acute lymphoblastic leukemia patients results in high response rate and prolonged leukemia-free survival. J. Clin. Oncol..

[25] Watanabe A., Inukai T., Kagami K., Abe M., Takagi M., Fukushima T., Fukushima H., Nanmoku T., Terui K., Ito T. (2019). Resistance of t(17;19)-acute lymphoblastic leukemia cell lines to multiagents in induction therapy. Cancer Med..

[26] Fischer U., Forster M., Rinaldi A., Risch T., Sungalee S., Warnatz H.J., Bornhauser B., Gombert M., Kratsch C., Stütz A.M. (2015). Genomics and drug profiling of fatal TCF3-HLF-positive acute lymphoblastic leukemia identifies recurrent mutation patterns and therapeutic options. Nat. Genet..

[27] Mouttet B., Vinti L., Ancliff P., Bodmer N., Brethon B., Cario G., Chen-Santel C., Elitzur S., Hazar V., Kunz J. (2019). Durable remissions in TCF3-HLF positive acute lymphoblastic leukemia with blinatumomab and stem cell transplantation. Haematologica.

