# Supplementary material for: Epigenetic Modification of Death Receptor Genes for TRAIL and TRAIL Resistance in Childhood B-Cell Precursor Acute Lymphoblastic Leukemia
Source: Genes (Basel). 2021 Jun 5;12(6):864. doi: 10.3390/genes12060864 (PMC8229974; doi:10.3390/genes12060864)
Supplement: Supplementary file 1 [file genes-12-00864-s001.zip › Supplementary Figures.pdf]

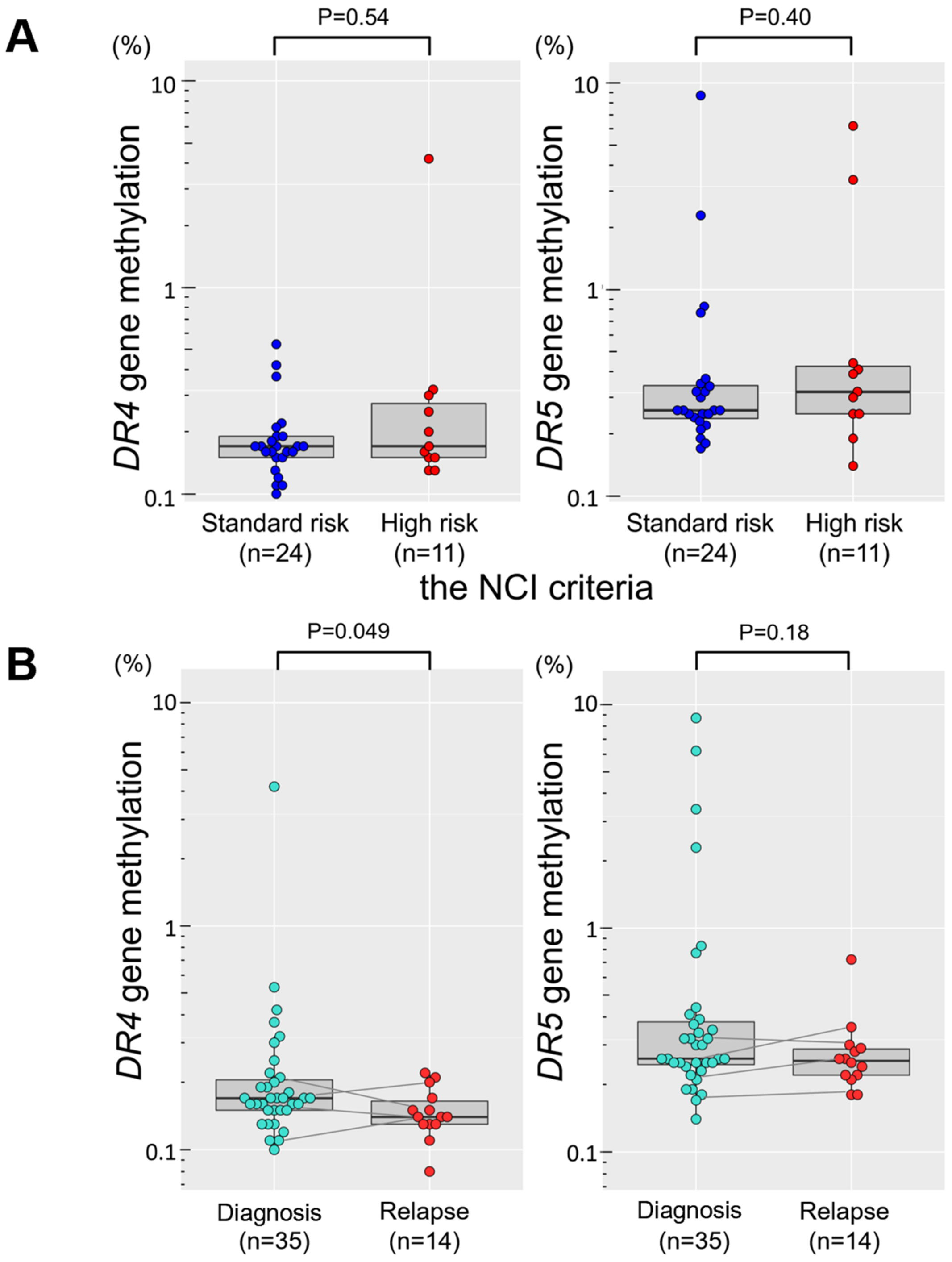

Supplementary Figure S1.

A, Comparison of the methylation status of the *DR4* and *DR5* genes between the BCP-ALL samples at diagnosis in NCI standard risk group (n=24) and those in high risk group (n=11). Vertical axes indicate a log_{10} percent methylation of the *DR4*(left panel) and

the *DR5*(right panel) genes. P-values in Mann-Whitney's U test are indicated at the top of the panel.

B, Comparison of the methylation status of the *DR4* and *DR5* genes between the BCP-ALL samples at diagnosis (n=35) and those at relapse (n=11). Vertical axes indicate a $\log_{10}$ percent methylation of the *DR4*(left panel) and the *DR5*(right panel) genes. Lines indicate the changes in the four paired samples. P-values in Mann-Whitney's U test are indicated at the top of the panel.

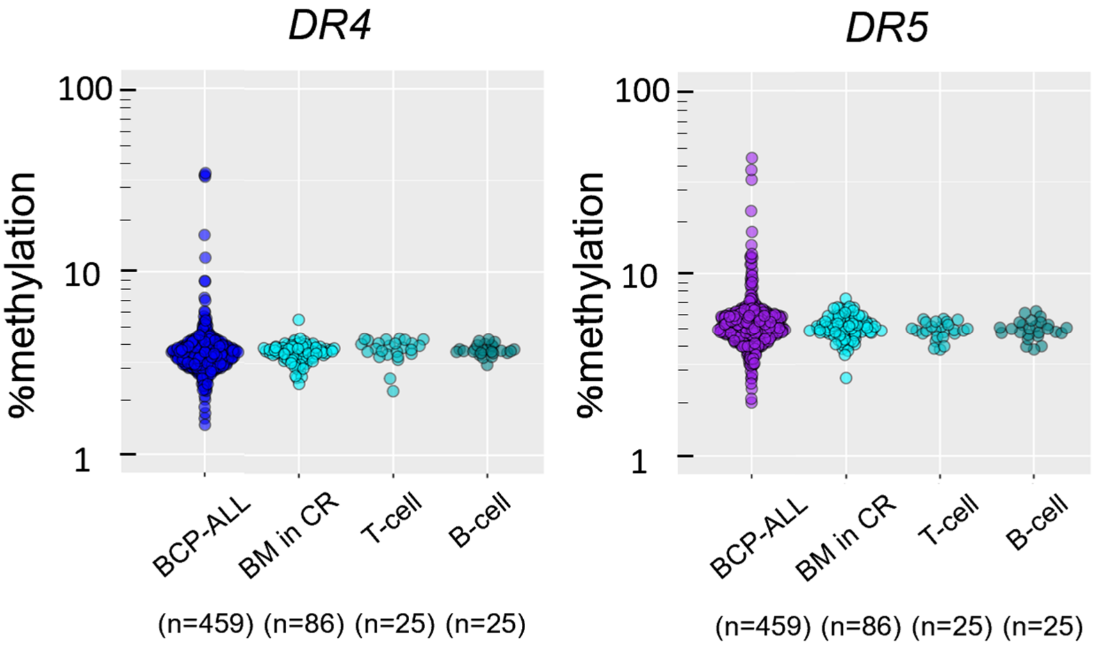

Supplementary Figure S2.

The *DR4* and *DR5* methylation status in BCP-ALL samples at diagnosis (n=459), bone marrow (BM) samples in complete remission (CR) (n=86), and normal peripheral T-cells (n=25) and B-cells (n=25) in the NOPHO database. Vertical axes indicate a $\log_{10}$ percent methylation of the *DR4* (left panel) and that of the *DR5* (right panel).
